# Immunohistochemical detection of epidermal growth factor receptors on human colonic carcinomas.

**DOI:** 10.1038/bjc.1990.63

**Published:** 1990-02

**Authors:** R. J. Steele, P. Kelly, B. Ellul, O. Eremin

**Affiliations:** Department of Surgery, University of Aberdeen, UK.


Br. J. Cancer (1990), 61, 325–326 © Macmillan Press Ltd., 1990

SHORT COMMUNICATION

### Immunohistochemical detection of epidermal growth factor receptors on human colonic carcinomas

R.J.C. Steele, P. Kelly, B. Ellul & O. Eremin

*Departments of Surgery and Pathology, University of Aberdeen, UK.*

Epidermal growth factor (EGF) and transforming growth factor α (TGFα) are peptide growth hormones which are known to regulate the proliferation of a variety of cells (Carpenter & Cohen, 1979; Cohen, 1983; Marquardt *et al.*, 1983) including the gastrointestinal cells in rodents (Al-Naffusi & Wright, 1982). Both of these peptides exert their influences via the EGF receptor, a 170 kDa phosphoglycoprotein located across the cell membrane (Carpenter & Cohen, 1979). The internal portion of this receptor bears a close relationship to the v-*erb*-B oncogene product (Downward *et al.*, 1984), and it is likely that growth factors play a role in oncogenesis (Burgess, 1985).

Certainly, a number of human neoplastic cell types have been shown to express the EGF receptor to a variable degree (Gusterson *et al.*, 1984), and there is evidence that increased expression is associated with a poor prognosis (Neal *et al.*, 1985; Sainsbury *et al.*, 1987). Little work has been done on colonic cancer, and although this tumour has been shown to carry the receptor (Bradley *et al.*, 1986; Yasui *et al.*, 1988), no firm association with prognostic factors has been established. The aim of this study was to evaluate different methods of immunohistochemical identification of EGF receptors on human colonic cancers, and to investigate the relationship between receptor expression and morphological differentiation.

In all, 30 colonic carcinomas were studied, a portion being taken immediately after resection and placed into liquid nitrogen. Frozen sections (6 μm) were then cut, air dried overnight, wrapped in aluminium foil and stored at −20°C. The primary antibody used was mouse monoclonal anti-human EGF receptor (Amersham) which is a class G2b immunoglobulin produced using trypsinised A431 cells as the immunogen (Waterfield *et al.*, 1982). This antibody recognises an antigenic determinant located on the extracellular domain of the receptor (Mayes & Waterfield, 1984) but does not compete with EGF for the ligand binding site (Gullick *et al.*, 1984).

In order to identify EGF receptors, three methods of immunoperoxidase staining were evaluated: indirect, peroxidase–antiperoxidase (PAP) and streptavidin–biotin (sABC) (Guesdon *et al.*, 1979; Polak & Van Noorden, 1983; Sternberger, 1979). The indirect method utilised peroxidase-conjugated rabbit anti-mouse immunoglobulin (Dakopatts) as the second stage. For PAP staining, rabbit anti-mouse or peroxidase-conjugated rabbit anti-mouse was followed by peroxidase–mouse anti-peroxidase complex (Dakopatts). Both of these systems were tried using single and double techniques. The sABC method employed biotin-conjugated rabbit anti-mouse immunoglobulin and streptavidin complexed to biotinylated peroxidase (Dakopatts).

Various concentrations of all reagents were tested in order to obtain optimum staining, the primary antibody being used at 1:25, 1:50, 1:100, 1:200 and 1:400, and sections were incubated at room temperature for 60 min. The rabbit anti-mouse immunoglobulin was absorbed with 10% human AB serum. Bound peroxidase was visualised using the diaminobenzamine/$H_2O_2$ reaction, and sections were counterstained with haematoxylin, dehydrated and mounted in DPX. Two methods were used to block endogenous peroxidase staining. The first consisted of periodic acid at 0.228% placed on to the slides for 45 s after air drying (Kelly *et al.*, 1987), and the second involved making up the secondary antibody in TBS containing glucose oxidase (1.5 u $ml^{-1}$) and glucose (0.2M) (Koller *et al.*, 1986).

Frozen sections of human placental tissue were used as positive controls, and negative controls consisted of human thyroid tissue (Damjanov *et al.*, 1986; Gusterson *et al.*, 1979). In addition, every study slide was accompanied by a substitution control in which the primary antibody was replaced by non-immune mouse IgG (Sigma). At histological examination, the intensity of peroxidase staining was graded as 0, + or + +, and the colonic tumours were classified as well/moderately or poorly differentiated. This was done by two independent observers, and the grading of peroxidase staining intensity was performed without knowledge of the degree of differentiation.

In all cases, background and non-specific staining was minimal, although endogenous peroxidase in granulocytes was encountered when blocking was not employed. The syncytiotrophoblast of the placental tissue stained positively using all techniques, but strongest staining was obtained using the sABC method. No staining of formalin-fixed placental tissue could be obtained by any means. In the colonic tumours, positive staining was only obtained using the double PAP system, with peroxidase-conjugated rabbit anti-mouse as the secondary antibody, and the sABC method (Table I). Of these two, the sABC technique gave more intense, clearer staining and was much less time consuming. The ideal dilutions were found to be 1:100 for the primary antibody and 1:200 for the biotinylated rabbit anti-mouse.

Twenty-seven out of the 30 colonic cancers stained for EGF receptor, and the staining was confined to the cytoplasm of the neoplastic epithelial cells within the tumour. Both the periodic acid and glucose oxidase methods were effective in blocking endogenous peroxidase, but periodic acid abolished all EGF receptor staining, even in placental tissue.

**Table I** Intensity of immunohistochemical staining for EGF receptor on human placenta and colonic carcinoma using a variety of methods

| | *Placenta* | *Colonic carcinoma* |
|---|---|---|
| Single indirect | + | 0 |
| Double indirect | + + | 0 |
| Single PAP | + + | 0 |
| Double PAP | + + | 0 |
| Single PAP + PCRAM | + + | 0 |
| Double PAP + PCRAM | + + | + |
| sABC | + + | + + |

Results are based on the optimal primary antibody dilution of 1:100. 0, no staining; +, weak staining; + +, strong staining. PAP, peroxidase–antiperoxidase; PCRAM, peroxidase-conjugated rabbit anti-mouse; sABC, streptavidin–biotin complex.

Correspondence: R.J.C. Steele, Department of Surgery, University Medical Buildings, Foresterhill, Aberdeen, UK.
Received 8 May 1989; and in revised form 25 September 1989.

The glucose oxidase technique did not affect the immunoreactivity of the EGF receptor on any of the sections. Intensity of staining was uniform throughout individual tumours, but it was significantly greater in the poorly differentiated tumours when compared with the well/moderately differentiated cancers (Table II).

In this study we have shown that EGF receptors can be identified on the neoplastic cells of invasive human colonic cancers using a well characterised commercially available mouse monoclonal antibody, and we found the sABC system to be the most suitable for this purpose. Less sensitive methods were inadequate, and this presumably relates to the low concentrations of receptor found in colonic carcinoma using radioligand binding (Yasui *et al.*, 1988).

EGF receptors have been demonstrated on a variety of human tissues (Damjanov *et al.*, 1986; Gusterson *et al.*, 1984) including squamous cell carcinoma (Ozanne *et al.*, 1986), and neoplasms of the lung (Veale *et al.*, 1987), breast (Sainsbury *et al.*, 1985*a*), bladder (Neale *et al.*, 1985) and stomach (Yasui *et al.*, 1988). In breast cancer, EGF receptor expression has been shown to be associated with an absence of oestrogen receptors, poor differentiation and increased risk of early recurrence and death (Sainsbury *et al.*, 1985*b*, *c*, 1987, 1988). Similarly, in bladder cancer EGF receptor positive tumours tend to be invasive and poorly differentiated (Neale *et al.*, 1985), and in stomach carcinoma early tumours are less likely to have receptors than advanced cancers (Yasui *et al.*, 1988).

**Table II** Relationship between intensity of immunoperoxidase staining for EGF receptor and histological differentiation in colonic carcinoma

| | Staining intensity | |
|---|---|---|
| | 0/+ | ++ |
| Moderately/well differentiated | 16 | 7 |
| Poorly differentiated | 0 | 7 |

$\chi^2 = 7.83$ (With Yates' correction); $P<0.01$. Difference in percentage of tumours exhibiting ++ staining = 69%, with a 95% confidence interval of 50–88%.

Two previous studies have demonstrated EGF receptors in colonic carcinoma (Bradley *et al.*, 1986; Yasui *et al.*, 1988). In the first of these, radioligand binding, Western transfer and indirect immunofluorescence were used, and moderately well differentiated tumour cell lines were found to express receptor activity to a greater extent than poorly differentiated lines. There was, however, no detailed information on naturally occurring tumours in this report (Bradley *et al.*, 1986). In the second study, no association between tumour grade and EGF receptor expression could be demonstrated, and only 77% of the tumours were receptor postive (Yasui *et al.*, 1988).

Clearly, there are contradictory findings regarding the detection and significance of EGF receptors on human tumours. This may indicate biological differences between different tumour types, but it may also be related to the use of antibodies which recognise different epitopes on the receptor. Our data suggest that, when a highly sensitive technique is used, the majority of colonic carcinomas can be shown to express EGF receptors and that there is an association between the degree of expression and histological differentiation. Further elucidation of the prognostic significance of this finding must await follow-up of larger numbers of patients.

This work was supported by grants from the Royal College of Surgeons of Edinburgh and the Grampian Health Board.

### References

AL-NAFUSSI, A.I. & WRIGHT, N.A. (1982). The effect of epidermal growth factor (EGF) on cell proliferation of the gastrointestinal mucosa in rodents. *Virchows Arch. Cell Pathol.*, **40**, 63.

BRADLEY, S.J., GARFINKLE, G., WALKER, E., SALEM, R., CHEN, L.B. & STEELE, G. (1986). Increased expression of the epidermal growth factor receptor on human colon carcinoma cells. *Arch. Surg.*, **121**, 1242.

BURGESS, A. (1985). Growth factors and oncogenes. *Immunol. Today*, **6**, 107.

CARPENTER, G. & COHEN, S. (1979). Epidermal growth factor. *Ann. Rev. Biochem.*, **48**, 193.

COHEN, S. (1983). The epidermal growth factor (EGF). *Cancer*, **51**, 1787.

DAMJANOV, I., MILDNER, B. & KNOWLES, B.B. (1986). Immunohistochemical localisation of the epidermal growth factor receptor in normal human tissues. *Lab. Invest.*, **55**, 588.

DOWNWARD, J., YARDEN, Y., MAYES, E. & 6 others (1984). Close similarity of epidermal growth factor receptor and v-*erb*-B oncogene protein sequences. *Nature*, **307**, 521.

GUESDON, J.L., TERNYCK, T. & AVRAMENS, S. (1979). The use of avidin-biotin interaction in immunoenzymatic techniques. *J. Histochem. Cytochem.*, **27**, 1131.

GULLICK, W.J., DOWNWARD, J.H., MARSDEN, J.J. & WATERFIELD, M.D. (1984). A radioimmunoassay for human epidermal growth factor receptor. *Anal. Biochem.*, **141**, 253.

GUSTERSON, B., COWLEY, G., SMITH, J.A. & OZANNE, B. (1984). Cellular localisation of human epidermal growth factor receptor. *Cell. Biol. Intern. Rep.*, **8**, 649.

KELLY, J., WHEELAN, C.D., WEIR, D.G. & FEIGHERY, C. (1987). Removal of endogenous peroxidase activity from cryostat sections for immunoperoxidase visualisation of monoclonal antibodies. *J. Immunol. Methods*, **96**, 127.

KOLLER, U., STOCKINGER, H., MAJDIC, O., BETTELHEIM, P. & KNAPP, W. (1986). A rapid and simple immunoperoxidase staining procedure for blood and bone marrow samples. *J. Immunol. Methods*, **86**, 75.

MARQUARDT, H., HUNKAPILLER, M.W., HOOD, L.F. & 4 others (1983). Transforming growth factors produced by retrovirus transformed rodent fibroblasts and human melanoma cells: amino acid sequence homology with epidermal growth factor. *Proc. Natl Acad. Sci. USA*, **80**, 4684.

MAYES, E.L.V. & WATERFIELD, M.D. (1984). Biosynthesis of the epidermal growth factor receptor in A431 cells. *EMBO J.*, **3**, 531.

NEAL, D.E., MARSH, C., BENNETT, M.K. & 4 others (1985). Epidermal growth factor receptors in human bladder cancer: comparison of invasive and superficial tumours. *Lancet*, **i**, 366.

OZANNE, B., RICHARDS, C.S., HENDLER, F., BURNS, O. & GUSTERSON, B. (1986). Over-expression of the EGF receptor is a hallmark of squamous cell carcinomas. *J. Pathol.*, **149**, 9.

POLAK, J.M. & VAN NOORDEN, S. (1983). *Immunocytochemistry. Practical Applications in Pathology and Biology.* Wright-PSG: Boston.

STERNBERGER, L.A. (1979). *Immunocyctochemistry*, 2nd edn. John Wiley & Sons: New York.

SAINSBURY, J.R.C., SHERBET, G.V., FARNDON, J.R. & HARRIS, A.L. (1985*a*). Epidermal growth factor receptors on human breast cancers. *Br. J. Surg.*, **72**, 186.

SAINSBURY, J.R.C., FARNDON, J.R., SHERBET, G.V. & HARRIS, A.L. (1985*b*). Epidermal growth factor receptors and oestrogen receptors in human breast cancer. *Lancet*, **i**, 364.

SAINSBURY, J.R.C., MALCOLM, A., APPLETON, D., FARNDON, J.R. & HARRIS, A.L. (1985*c*). Presence of epidermal growth factor receptors as an indicator of poor prognosis in patients with breast cancer. *J. Clin. Pathol.*, **38**, 1225.

SAINSBURY, J.R.C., NEEDHAM, G.K., MALCOLM, A., FARNDON, J.R. & HARRIS, A.L. (1987). Epidermal growth factor as predictor of early recurrence and death from breast cancer. *Lancet*, **i**, 1398.

SAINSBURY, J.R.C., NICHOLSON, S., ANGUS, B., FARNDON, J.R., MALCOLM, A.J. & HARRIS, A.L. (1988). Epidermal growth factor receptor status of histological sub-types of breast cancer. *Br. J. Cancer*, **58**, 458.

WATERFIELD, M.D., MAYES, E.L.B., STROOBANT, P. & 5 others (1982). A monoclonal antibody to the human epidermal growth factor. *J. Cell. Biol.*, **20**, 149.

VEALE, D., ASHCROFT, T., MARSH, C., GIBSON, G.J. & HARRIS, A.L. (1987). Epidermal growth factor receptors in non-small cell lung cancer. *Br. J. Cancer*, **55**, 513.

YASUI, W., SUMIYOSHI, H., HATA, J. & 4 others (1988). Expression of epidermal growth factor receptor in human gastric and colonic carcinomas. *Cancer Res.*, **48**, 137.


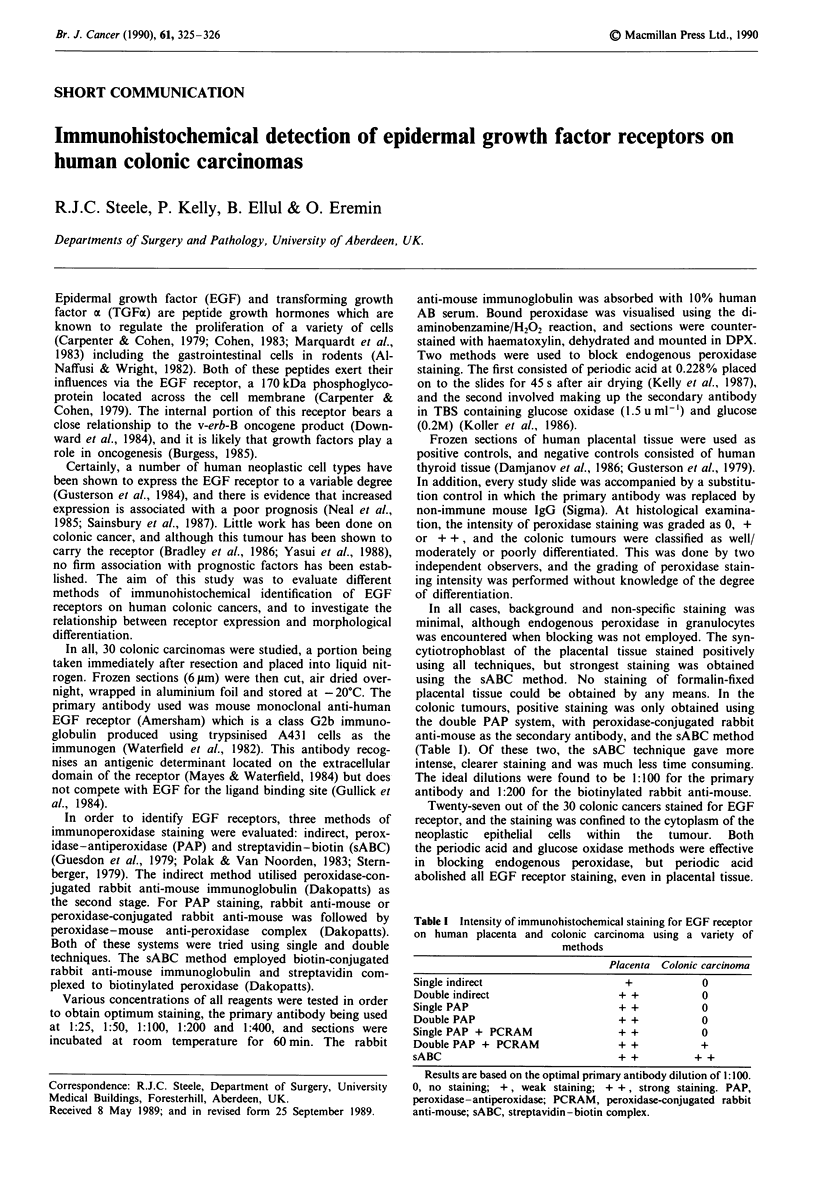

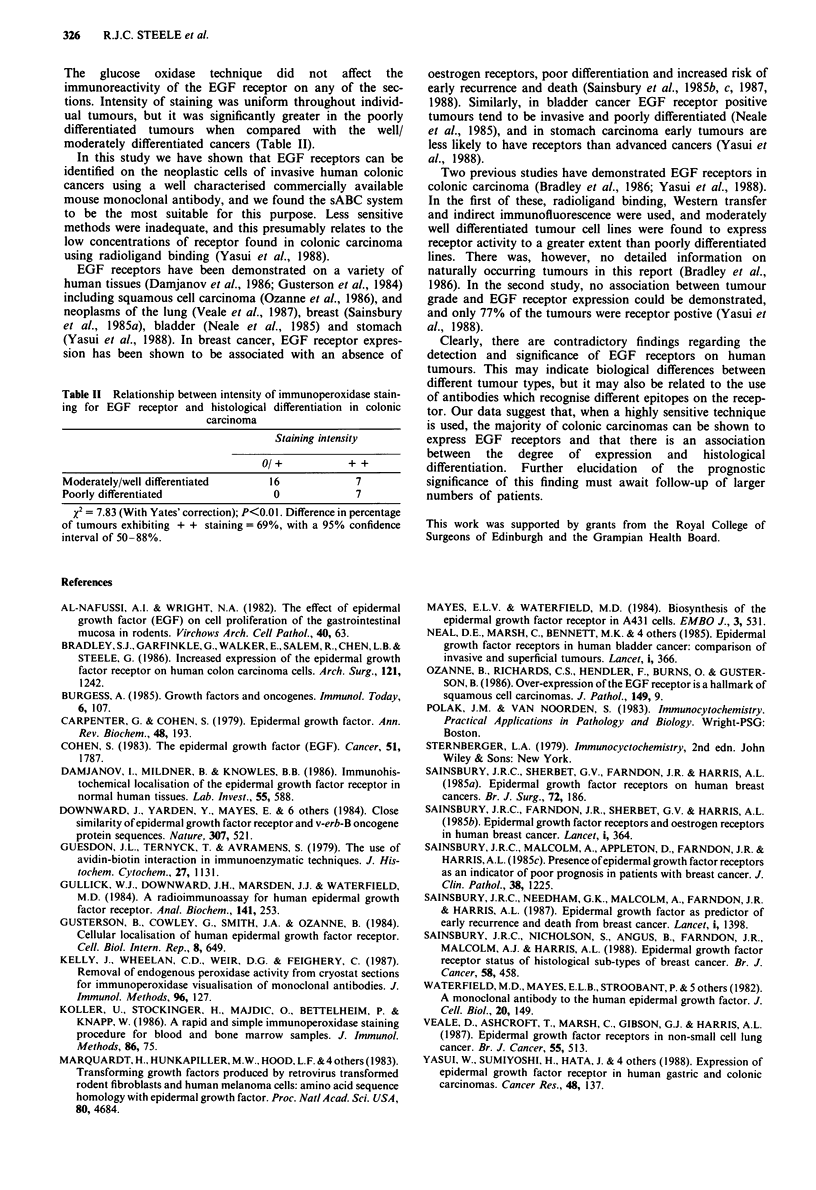


## References

[OCR_00226] Al-Nafussi A. I., Wright N. A. (1982). The effect of epidermal growth factor (EGF) on cell proliferation of the gastrointestinal mucosa in rodents.. Virchows Arch B Cell Pathol Incl Mol Pathol.

[OCR_00231] Bradley S. J., Garfinkle G., Walker E., Salem R., Chen L. B., Steele G. (1986). Increased expression of the epidermal growth factor receptor on human colon carcinoma cells.. Arch Surg.

[OCR_00241] Carpenter G., Cohen S. (1979). Epidermal growth factor.. Annu Rev Biochem.

[OCR_00245] Cohen S. (1983). The epidermal growth factor (EGF).. Cancer.

[OCR_00249] Damjanov I., Mildner B., Knowles B. B. (1986). Immunohistochemical localization of the epidermal growth factor receptor in normal human tissues.. Lab Invest.

[OCR_00254] Downward J., Yarden Y., Mayes E., Scrace G., Totty N., Stockwell P., Ullrich A., Schlessinger J., Waterfield M. D. (1984). Close similarity of epidermal growth factor receptor and v-erb-B oncogene protein sequences.. Nature.

[OCR_00259] Guesdon J. L., Ternynck T., Avrameas S. (1979). The use of avidin-biotin interaction in immunoenzymatic techniques.. J Histochem Cytochem.

[OCR_00264] Gullick W. J., Downward D. J., Marsden J. J., Waterfield M. D. (1984). A radioimmunoassay for human epidermal growth factor receptor.. Anal Biochem.

[OCR_00269] Gusterson B., Cowley G., Smith J. A., Ozanne B. (1984). Cellular localisation of human epidermal growth factor receptor.. Cell Biol Int Rep.

[OCR_00274] Kelly J., Whelan C. A., Weir D. G., Feighery C. (1987). Removal of endogenous peroxidase activity from cryostat sections for immunoperoxidase visualisation of monoclonal antibodies.. J Immunol Methods.

[OCR_00280] Köller U., Stockinger H., Majdic O., Bettelheim P., Knapp W. (1986). A rapid and simple immunoperoxidase staining procedure for blood and bone marrow samples.. J Immunol Methods.

[OCR_00286] Marquardt H., Hunkapiller M. W., Hood L. E., Twardzik D. R., De Larco J. E., Stephenson J. R., Todaro G. J. (1983). Transforming growth factors produced by retrovirus-transformed rodent fibroblasts and human melanoma cells: amino acid sequence homology with epidermal growth factor.. Proc Natl Acad Sci U S A.

[OCR_00293] Mayes E. L., Waterfield M. D. (1984). Biosynthesis of the epidermal growth factor receptor in A431 cells.. EMBO J.

[OCR_00296] Neal D. E., Marsh C., Bennett M. K., Abel P. D., Hall R. R., Sainsbury J. R., Harris A. L. (1985). Epidermal-growth-factor receptors in human bladder cancer: comparison of invasive and superficial tumours.. Lancet.

[OCR_00303] Ozanne B., Richards C. S., Hendler F., Burns D., Gusterson B. (1986). Over-expression of the EGF receptor is a hallmark of squamous cell carcinomas.. J Pathol.

[OCR_00315] Sainsbury J. R., Farndon J. R., Harris A. L., Sherbet G. V. (1985). Epidermal growth factor receptors on human breast cancers.. Br J Surg.

[OCR_00331] Sainsbury J. R., Farndon J. R., Needham G. K., Malcolm A. J., Harris A. L. (1987). Epidermal-growth-factor receptor status as predictor of early recurrence of and death from breast cancer.. Lancet.

[OCR_00336] Sainsbury J. R., Nicholson S., Angus B., Farndon J. R., Malcolm A. J., Harris A. L. (1988). Epidermal growth factor receptor status of histological sub-types of breast cancer.. Br J Cancer.

[OCR_00347] Veale D., Ashcroft T., Marsh C., Gibson G. J., Harris A. L. (1987). Epidermal growth factor receptors in non-small cell lung cancer.. Br J Cancer.

[OCR_00342] Waterfield M. D., Mayes E. L., Stroobant P., Bennet P. L., Young S., Goodfellow P. N., Banting G. S., Ozanne B. (1982). A monoclonal antibody to the human epidermal growth factor receptor.. J Cell Biochem.

[OCR_00352] Yasui W., Sumiyoshi H., Hata J., Kameda T., Ochiai A., Ito H., Tahara E. (1988). Expression of epidermal growth factor receptor in human gastric and colonic carcinomas.. Cancer Res.

